# Toxicological Evaluation of *Zingiber purpureum* Extract and Its Effects on Gut Microbiota in Zebrafish

**DOI:** 10.3390/ijms27198684

**Published:** 2026-09-28

**Authors:** Nisamanee Charoenchon, Porntipa Korprasertthaworn, Jitima Thammavoharnkul, Nattanan Kiatsurayanon, Phannamon Prathomthong, Donruethai Chimmanee, Athisri Sitthipunya

**Affiliations:** 1 Department of Pathobiology, Faculty of Science, Mahidol University, Bangkok 10400, Thailand; nisamanee.cha@mahidol.ac.th (N.C.); nattanan.kia@alumni.mahidol.ac.th (N.K.); phannamon.prt@student.mahidol.edu (P.P.); donruethai.chm@student.mahidol.edu (D.C.); 2 Zebrafish-Based Research in Aging Biology and Microbiome (ZAM) Laboratory, Department of Pathobiology, Faculty of Science, Mahidol University, Bangkok 10400, Thailand; 3 Department of Pharmacology, Faculty of Science, Mahidol University, Bangkok 10400, Thailand; porntipa.kor@mahidol.ac.th; 4 Graduate Program in Toxicology, Faculty of Science, Mahidol University, Bangkok 10400, Thailand; jitima.tha@student.mahidol.ac.th

**Keywords:** *Zingiber purpureum* Roscoe, DMPBD, curcumin, embryotoxicity, gut microbiome, zebrafish

## Abstract

*Zingiber purpureum* Roscoe (Phlai) is renowned in Southeast Asia for its anti-inflammatory, antioxidant, and antibacterial activities, yet its safety and effects on gut health in vivo are not well characterized. This study investigated the biological effects of ethanolic-soluble Phlai extract (ESPE) in zebrafish (*Danio rerio*), focusing on chemical marker standardization in phenylbutanoids and cucurminoids, developmental toxicity, gut microbiota investigation, and tissue histopathology. Zebrafish embryos were exposed to ESPE to assess toxicity while adult females received an ESPE-enriched diet containing 20 mg ESPE/g diet (2% *w*/*w*) for 14 days. Post-treatment analyses included intestinal and multi-organ histology, and 16S rRNA sequencing for microbiome profiling. HPLC analysis confirmed that ESPE contained 9.91% (*w*/*w*) of (E)-1-(3,4-dimethoxyphenyl)butadiene (DMPBD) and 0.49% (*w*/*w*) curcumin. The 96-h LC50 for embryos was 45.54 µg/mL. In adults, ESPE improved gut health, as shown by greater mucosal thickness, increased goblet cells, and enhanced enterocyte organization. Microbiome analysis showed that overall gut microbial alpha diversity and beta diversity did not differ significantly between groups. However, exploratory differential abundance analysis identified differences in the relative abundance of specific genera in the ESPE-treated group, including *Crenobacter*, *Cellvibrio*, *Pseudaeromonas*, and *Starkeya*. Muscle tissue demonstrated healthy myofibril regeneration without fibrosis. ESPE was well tolerated, enhanced intestinal function, and without systemic toxicity. These findings support the potential of ESPE as a bioactive extract for further investigation of intestinal health and safety in translational pharmacology applications.

## 1. Introduction

**Figure 1:**
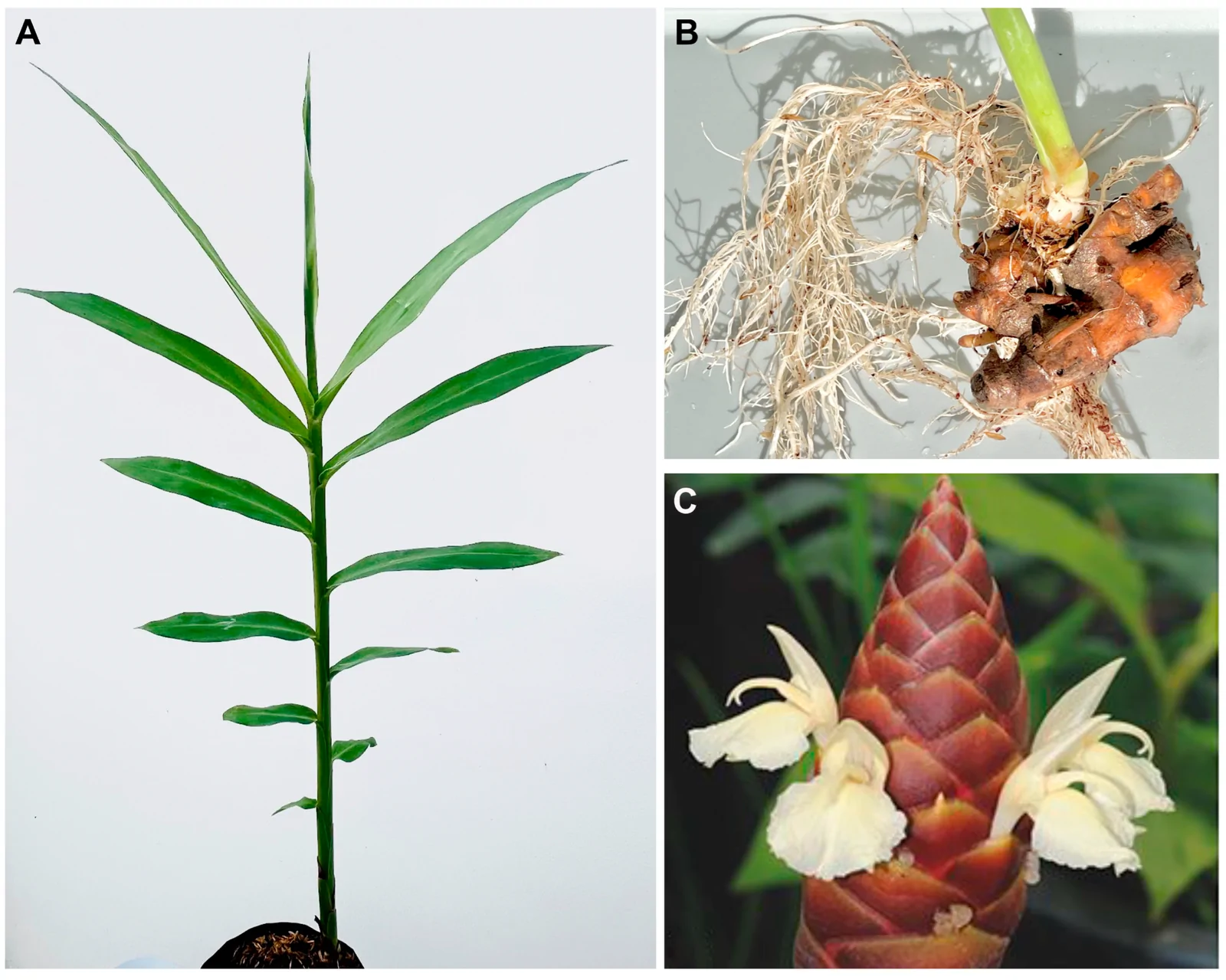
Morphological characteristics of *Zingiber purpureum* Roscoe (Phlai). (**A**) Whole plant. (**B**) Rhizome and fibrous roots. (**C**) Inflorescence showing reddish-brown bracts and cream-white flowers. Panels (**A**,**B**) were photographed from the plant specimen used in this study. Panel (**C**) was adapted from the Flora of Thailand database (Forest Herbarium, Department of National Parks, Wildlife and Plant Conservation, Thailand).

*Zingiber purpureum* Roscoe (*Z. purpureum*) is a prominent medicinal ginger species in Southeast Asia, where it is widely known as Phlai or Plai [1,2,3]. Taxonomically, it belongs to the Zingiberaceae family [4]. The species is characterized by distinctive morphological features, including dark red, purplish, or brown pubescent bracts and cream-white inflorescences, as shown in Figure 1.

Traditionally, Phlai rhizomes have been used as local remedies, applied topically to treat skin diseases, infections, and open wounds [5,6]. Recent research has also revealed that these rhizomes contain a diverse array of bioactive compounds, particularly phenylbutenoids [1,7,8]. The use of *Z. purpureum* for skin infections is now supported by scientific evidence showing that its essential oil exhibits marked antibacterial activity, making it a viable candidate for use in wound-dressing materials [6]. To validate these traditional dermatological practices, recent research has employed internationally standardized methodologies. Pharmacological studies have underscored the biomedical relevance of this species; for instance, *Z. purpureum* Roscoe powders have been shown to exhibit high antioxidant activity and pronounced antimicrobial effects against a range of pathogenic microorganisms, attributes linked to its unique phytochemical composition [2]. Regarding skin inflammation, Phlai’s long-standing use in traditional medicine to soothe localized irritation has been mechanistically elucidated by modern research, which identifies inhibition of key inflammatory mediators, such as cyclooxygenase-2 (COX-2), as a principal mechanism [8,9]. Furthermore, recent studies highlight the potential of *Z. purpureum* essential oils as natural antibacterial agents for acne therapy and skincare product development [2], a finding corroborated by clinical trials demonstrating efficacy in alleviating muscle pain and myofascial discomfort [3]. Additionally, the use of boiled *Z. purpureum* rhizome powder by folk practitioners to manage hypertension and promote circulatory health is officially recognized by the Thai traditional medicine system under the Ministry of Public Health [3,10,11]. It was incorporated into polyherbal oral formulations with pepper, clove, and borneol and prepared as hot-water infusions or compressed herbal pills for gastrointestinal, respiratory, and musculoskeletal ailments [12]. Oral dietary supplementation with *Z. purpureum* extract may also help prevent age-related cognitive decline by promoting hippocampal neurogenesis and improving learning and memory [13]. One-month dietary supplementation with *Z. purpureum* extract improved cognitive function and hippocampal neurogenesis in an accelerated-aging mouse model [14]. The extracts have also been developed as an oral preparation for their neurotrophic properties, promoting hippocampal neuron regeneration, and have demonstrated safety in both animal toxicity studies and short-term human clinical trials [15]. In mice, oral administration of *Z. purpureum* rhizome extract improved DSS-induced colitis by suppressing inflammatory responses and modulating the AMPK/mTOR/NFκB signaling pathway [16] and by reducing intestinal inflammation and suppressing NFκB-related inflammatory responses [17].

The gut microbiome constitutes a highly complex community of microbes in the intestinal tract of animals [18,19,20,21]. This gut microbial community plays an important role in host metabolic processes essential to health, including nutrient digestion and absorption [22,23,24], immune system modulation [25,26,27], and protection against pathogens [26,28]. Several studies have reported factors that influence the gut microbial composition and function, including host genetics [18,29], age [18,30], diet [18,31,32], and environmental exposures [18,33]. Among those factors, dietary intake and oral supplementation serve as key substrates that support microbial fermentation and growth, thereby sustaining community stability [34,35]. Consequently, alterations in gut microbial composition and diversity often reflect and impact the host’s health status and immune function [18,27,32]. In the context of herbal medicine, the rhizomes of Phlai have demonstrated significant anti-inflammatory and antioxidant activities following oral administration of their extracts [2,8,9,36]. Building on these observations, the present study aims to evaluate the modulatory effects of orally administered herbal extract compounds on the gut microbiome. Zebrafish (*Danio rerio*) are employed as a model organism to assess the impact of these compounds on gut microbial composition and intestinal tissue integrity during administration [28,37].

Zebrafish were chosen as the in vivo model for studying the bioactive compound because they develop rapidly, have transparent embryos, and share approximately 70% genetic and physiological similarity with humans. They are also widely used in zebrafish embryotoxicity tests (ZET) [38] and embryo acute toxicity (FET) tests [39] to assess safety, survival rates, cardiotoxicity, and morphological defects in herbal extracts and chemical compounds. At the same developmental stage, other abnormalities, such as jaw deformity, liver toxicity, and neurotoxicity, can also be detected clearly [40]. Zebrafish also have an intestinal immune system that responds to dietary interventions similarly to humans, making them ideal for studying extract-induced gut dysbiosis [41,42]. Guided by these advantages and ethical principles, this project will evaluate the toxicological and developmental effects of ESPE in a zebrafish model.

Phlai extract was found to be extensively anti-inflammatory in the intestinal tracts of a mouse model. However, information on Phlai extract as a modulator of the gut microbiome is sparse. In our study, we not only evaluate the toxicological and developmental effects of ESPE on zebrafish but also novelly examine ESPE’s effects on the gut microbiome and intestinal parameters in a zebrafish model. We use an integrated approach combining microbiome analysis, histopathological parameter assessment, and bioactive compound assessment. We provide comprehensive insights into the safety and gut-modulatory potential of Phlai extract, focusing on its microbiome, thereby informing its biomedical applications.

## 2. Results

### 2.1. Developmental Toxicity and Morphological Abnormalities of Zebrafish Embryos Exposed to ESPE

**Figure 2:**
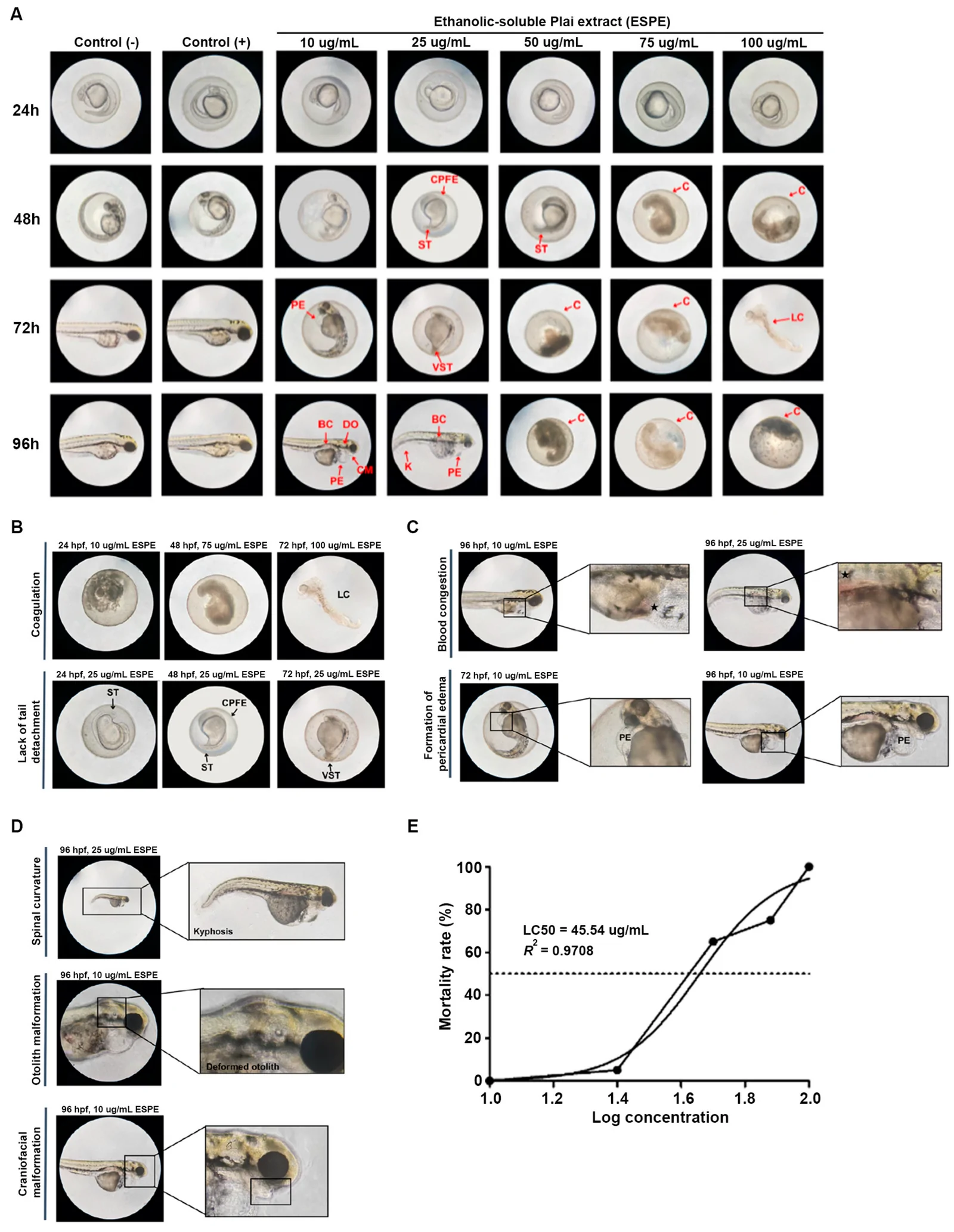
Developmental toxicity and morphological abnormalities in zebrafish (*Danio rerio*) embryos exposed to various concentrations of ethanolic-soluble Phlai extract (ESPE) from 24 to 96 h post-fertilization (hpf). (**A**) Spatiotemporal phenotypic profiling, representative matrix showing the developmental progression and induced malformations across different concentrations (10–100 μg/mL) and time points (24–96 h). Negative control, Control (−), and positive control, Control (+), are provided for comparison. Red abbreviations indicate specific observed endpoints: BC, blood congestion; C, coagulation; CM, craniofacial malformation; CPFE, collapse phenomenon of fertilized egg; DO, deformed otolith; K, kyphosis; LC, late coagulation; PE, pericardial edema; ST, short tail; VST, very short tail. (**B**) Core endpoints of lethality. Specific lethal phenotypes observed under OECD Test Guideline 236 conditions, demonstrating coagulation (e.g., at 24 hpf, 10 μg/mL; 48 hpf, 75 μg/mL; 72 hpf, 100 μg/mL) and lack of tail detachment/short tail development (e.g., at 25 μg/mL ESPE across 24–72 hpf). (**C**) Common sublethal endpoints with potential for regeneration. Mild-to-moderate malformations, including localized blood congestion (indicated by black asterisks) and initial formation of pericardial edema (PE), were observed primarily at lower concentrations (10–25 μg/mL) at 72 and 96 hpf. (**D**) Frequent sublethal observations with little or no potential for regeneration. Severe structural abnormalities induced by ESPE exposure at 96 hpf, including spinal curvature (kyphosis), otolith malformation (deformed otolith), and craniofacial malformation. All images were captured using a 10× objective lens on an inverted microscope. (**E**) Quantitative assessment of the median lethal concentration (LC50) of ESPE in zebrafish embryos at 96 hpf. The non-linear regression sigmoidal dose–response curve models the dynamic relationship between log-transformed ESPE concentrations and cumulative embryonic mortality (%).

The evaluation of zebrafish embryos from 24 to 96 hpf demonstrates that the extract induces profound dose- and time-dependent toxicity, disrupting normal embryonic development. The progression of developmental defects is strongly correlated with both increasing ESPE concentrations (10–100 µg/mL) and exposure duration, as shown in Figure 2A, revealing the spatiotemporal phenotypic profiling. While the negative control group exhibits normal, unhindered development, the ESPE-treated embryos display a spectrum of anomalies. Higher concentrations accelerate the onset of severe, acute phenotypes, whereas lower doses elicit progressive sublethal malformations over time.

According to the OECD Test Guideline 236 criteria, ESPE exposure triggered definitive lethal phenotypes, as shown in Figure 2B: the core endpoints of lethality. Embryonic death is characterized by protein coagulation. This lethal effect is observed at concentrations as low as 10 µg/mL during the early 24 hpf stage and culminates in late-stage coagulation and total egg collapse at higher concentrations (75–100 µg/mL) at later time points. In addition, a distinct failure in embryonic detachment, where the tail fails to separate from the yolk sac alongside severely stunted tail growth, is persistently observed from 24 to 72 hpf, even at a moderate concentration of 25 µg/mL. At the lower concentration spectrum (10–25 µg/mL), the common sublethal endpoints with regenerative potential are shown in Figure 2C. The embryos survived but developed mild-to-moderate circulatory disturbances by 72 and 96 hpf. These anomalies include localized blood congestion and the early-stage formation of pericardial edema. Because these tissue disruptions occur at a sub-critical threshold, they represent transient injuries that the embryos could potentially recover from or regenerate. By 96 hpf, the permanent structural malformations (with little to no regenerative potential) shown in Figure 2D result from prolonged exposure to ESPE and lead to irreversible, severe skeletal and sensory defects with virtually no potential for regeneration. The structural integrity of the larvae is severely compromised, as evidenced by pronounced spinal curvature (kyphosis) and craniofacial malformations. Additionally, the otic system is disrupted, with deformed otoliths that likely compromise the larvae’s balance and acoustic signaling.

To quantitatively evaluate the acute embryotoxic potency of ESPE, the median lethal concentration (LC50) was determined at the definitive 96 hpf endpoint. Nonlinear regression analysis with a variable slope sigmoid model was applied to the concentration-mortality data, establishing a highly robust correlation between ESPE exposure thresholds and lethal outcomes (*R*^2^ = 0.9708). As illustrated in Figure 2E, the cumulative embryonic mortality followed a classic sigmoidal, dose-dependent trajectory. The calculated 96 h LC50 value for ESPE in zebrafish embryos was established at 45.54 μg/mL (95% confidence interval: 34.29 to 60.48 μg/mL), corresponding to a log concentration of approximately 1.66. Minimal mortality was observed at the lowest tested log concentrations of 1.0 to 1.4 (equivalent to 10–25 μg/mL). However, a sharp inflection point in the sigmoidal curve occurred beyond this threshold, where the mortality rate escalated abruptly, reaching near-total lethality at the highest log concentration of 2.0 (equivalent to 100 μg/mL). This quantitative lethal threshold directly bridges the transition from reversible sublethal anomalies to widespread systemic failure and protein coagulation observed in the morphological assessment, establishing strict safety boundaries for the bioactive application of ESPE. The quantitative LC50 graph is highly consistent with the morphological observations in Figure 2A–D. The calculated LC50 value of 45.54 μg/mL is strongly supported by the phenotypic alterations observed in Figure 2A–D, where a clear transition from mild, reversible sublethal effects (at 10–25 μg/mL) to severe systemic malformations (at 50 μg/mL) and ultimate embryonic coagulation (at 75–100 μg/mL) was concentration-dependently demonstrated.

### 2.2. Histological Evaluation of Adult Zebrafish

#### 2.2.1. Skeletal Muscle Tissue

**Figure 3:**
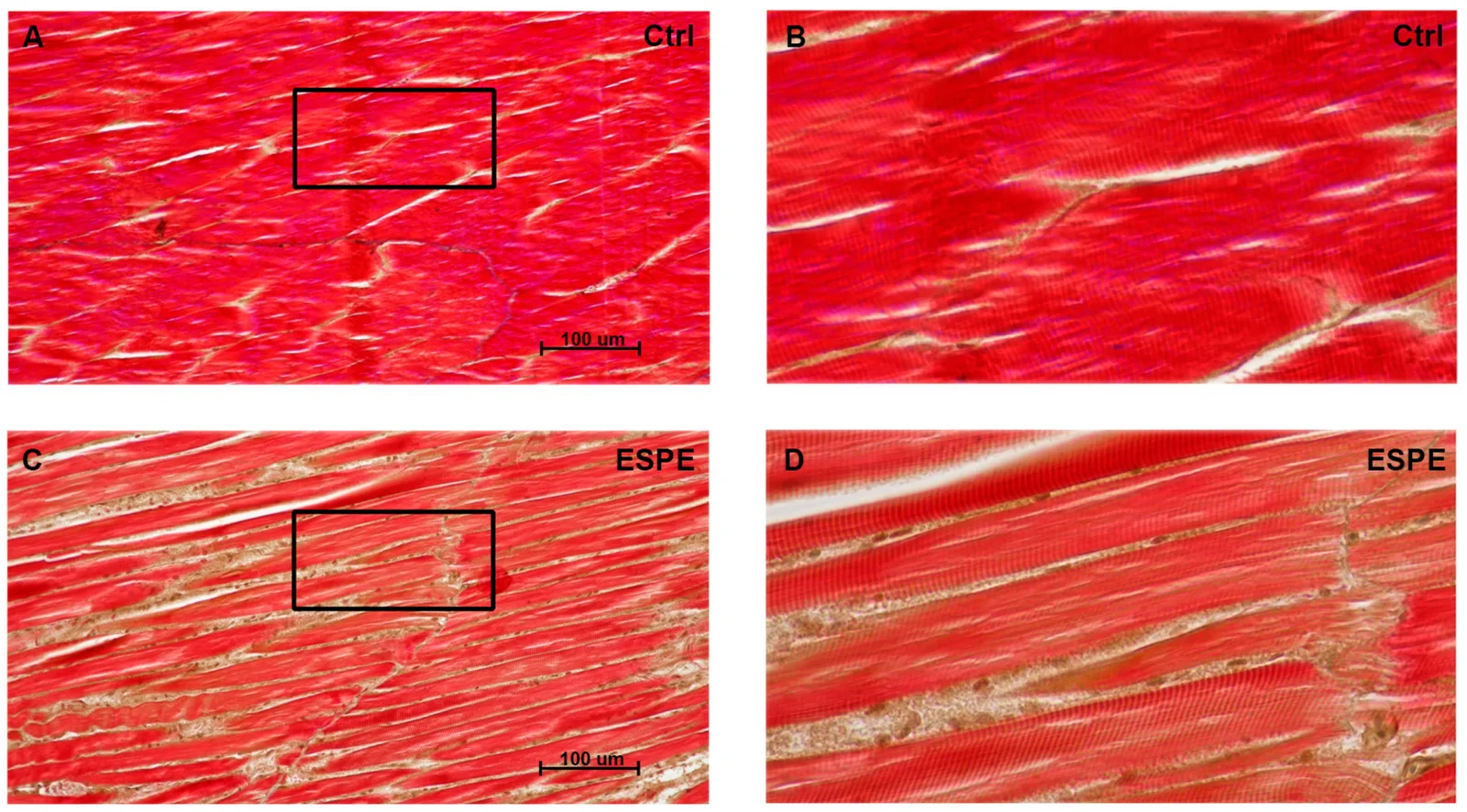
Histological comparison of skeletal muscle tissue between the control group and ESPE-treated adult zebrafish stained with Masson’s Trichrome. (**A**) Control group (Ctrl) exhibiting normal muscle fiber architecture at a lower 20× magnification (scale bar = 100 μm). (**B**) High-magnification view (40×) of the control group, representing the magnified area outlined by the boxed region in (**A**). (**C**) ESPE-treated group displaying muscle tissue morphology at a lower 20× magnification (scale bar = 100 μm). (**D**) High-magnification view (40×) of the ESPE-treated group, representing the magnified area outlined by the boxed region in (**C**), for detailed structural comparison. Ctrl: control group; ESPE: adult zebrafish fed an ESPE.-enriched diet containing 20 mg ESPE/g (2% *w*/*w*).

Histological analysis of adult zebrafish skeletal muscle tissue stained with Masson’s Trichrome, which differentiates muscle bundles in red and no collagen fibers (in green), revealed that the ESPE-treated group exhibited a normal and well-organized myofibril architecture. The muscle tissue maintained intact cellular boundaries with a typical muscle bundle and alignment along the longitudinal striations. Notably, the treatment appeared to induce a more juvenile or regenerative muscle profile, evidenced by an increased number of smaller-caliber muscle fibers, demonstrating not only well-preserved structural integrity but also improved muscle quality comparable to the control group, as shown in Figure 3.

#### 2.2.2. Skin

**Figure 4:**
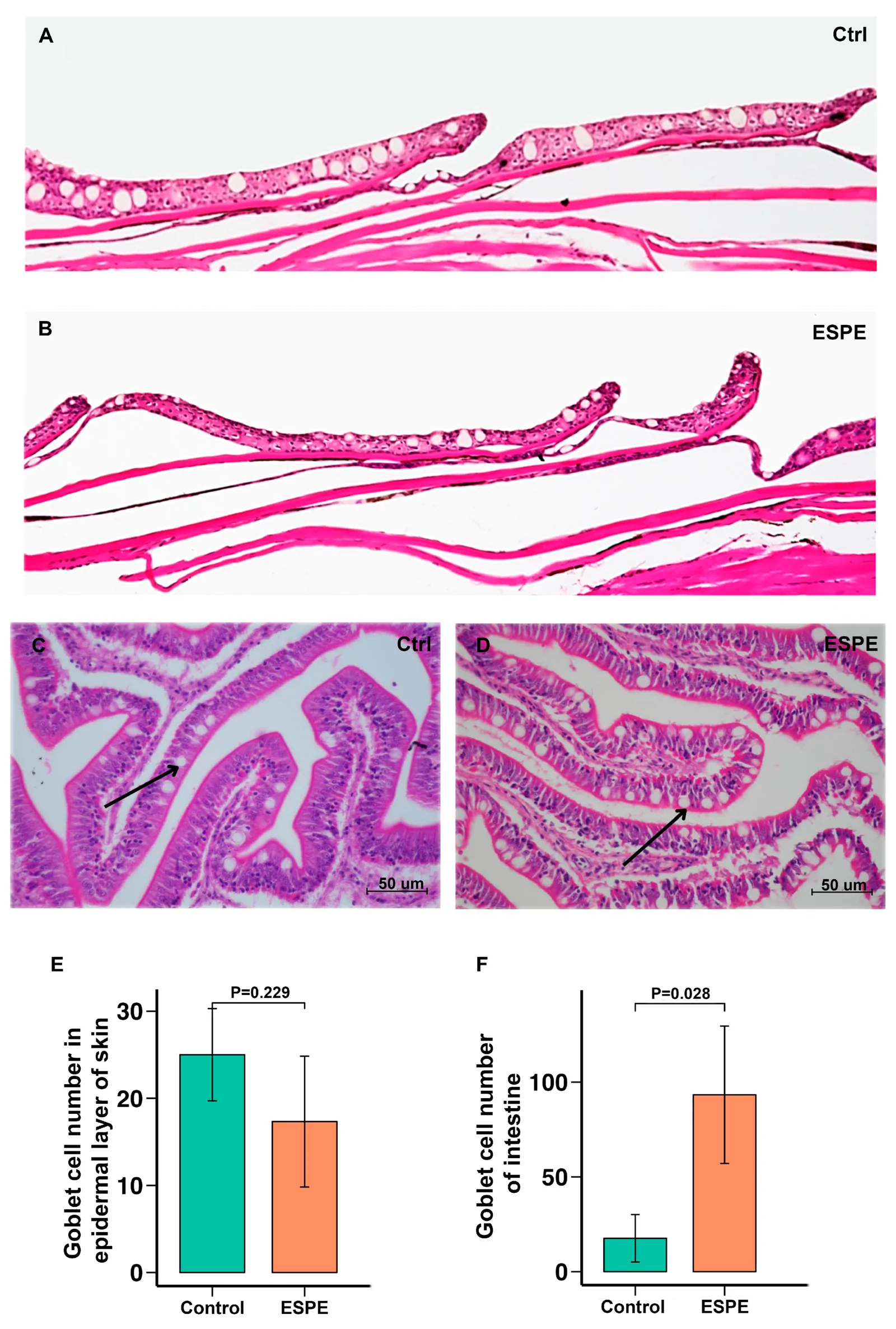
Histological evaluation of adult zebrafish skin and intestine by H&E staining. (**A**,**B**) Representative histology of skin from the control group (**A**) and the ESPE-treated group (**B**). (**C**,**D**) Representative histology of the intestine from the control group (**C**) and the ESPE-treated group (**D**). (**E**,**F**) Quantification and comparison of goblet cell numbers in the skin epithelium (**E**) and intestine (**F**) between the control (*n* = 3) and ESPE-treated groups (*n* = 3). The black arrow indicates the goblet cells in intestinal tissue. All images were captured at 10× magnification with a light microscope for comparative analysis. Ctrl: control group; ESPE: adult zebrafish fed an ESPE-enriched diet containing 20 mg ESPE/g (2% *w*/*w*).

Histological analysis of adult zebrafish skin structure via hematoxylin and eosin (H&E) staining demonstrated distinct morphological profiles between the groups (Figure 4A,B. Qualitative observation of the ESPE-treated group (Figure 4B) revealed noticeable architectural shifts, particularly marked by a visible change in epidermal thickness while maintaining overall structural framework without signs of severe detachment or lesions. However, quantitative evaluation of skin epidermal goblet cell density (Figure 4E) revealed no statistically significant difference between the control and ESPE-treated groups (*p* = 0.229).

#### 2.2.3. Intestine

Histological analysis was performed to compare intestinal morphology between control and ESPE-treated groups (Figure 4C,D). The analysis examined goblet cells, enterocytes, paneth cells, and immune-related cells, such as plasma cells and macrophages. In the control group (Figure 4C), intestinal folds were observed but appeared irregular in height and arrangement. The epithelium mainly consisted of enterocytes interspersed with relatively few goblet cells. Enterocytes exhibited typical columnar morphology with basally located nuclei, indicating healthy absorptive function. Mild epithelial flattening in some regions suggested potential stress or reduced mucosal renewal. In contrast, the ESPE-treated group (Figure 4D) showed more uniform, elongated intestinal folds and a smoother, more organized epithelial lining. Mononuclear cell infiltration was observed in the lamina propria of both groups, likely representing plasma cells or macrophages. Such immune cells are typical in healthy intestines and play a key role in mucosal immunity. The treated group exhibited a modestly higher density of these cells, but without clear signs of inflammation, indicating an adaptive mucosal immune response to the treatment rather than pathological infiltration. The mucosal architecture was better preserved, and a marked increase in goblet cells contributed to a visibly thicker mucosal layer. Quantification of goblet cells in the intestine (Figure 4F) revealed a significant increase in the ESPE-treated group compared to controls (*p* = 0.028).

### 2.3. Quantification of DMPBD and Curcumin in Phlai Crude Extract

**Figure 5:**
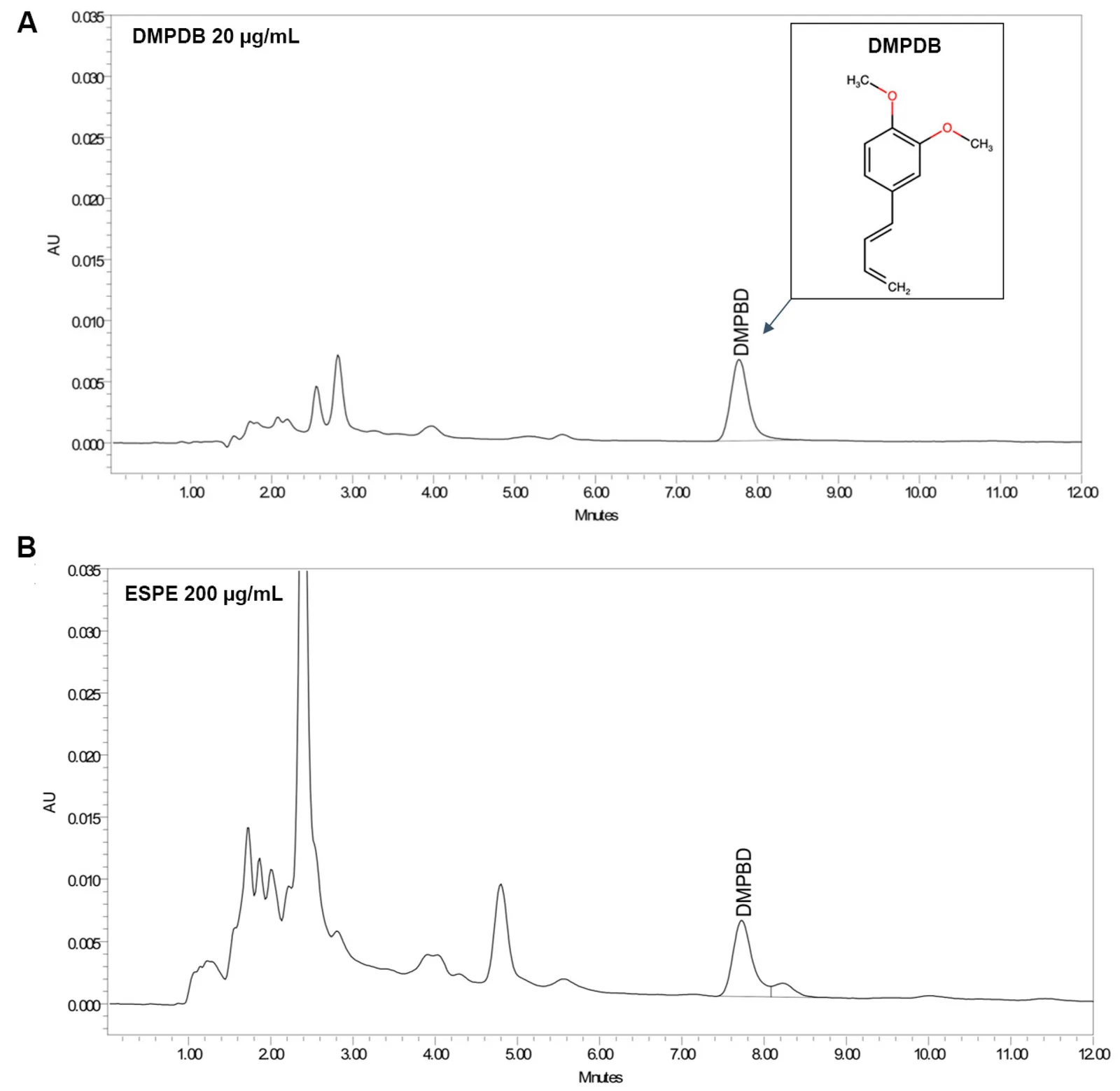
Representative HPLC chromatograms of (**A**) DMPBD reference standard (20 µg/mL) and (**B**) ESPE sample (200 µg/mL). Chromatographic separation was performed on a SunFire C18 column under isocratic conditions using acetonitrile and 2% aqueous acetic acid (52:48, *v*/*v*) as the mobile phase at a flow rate of 1.0 mL/min, with UV detection at 284 nm.

The HPLC chromatogram of the DMPBD (Figure 5) reference standard (20 µg/mL) showed a retention time (*t*_R_) of approximately 7.8 min (Figure 5). The calibration curve for DMPBD was constructed using standard solutions over the concentrations range of 0–80 µg/mL and showed good linearity, with a coefficient of determination (*R*^2^) of 0.99. The ESPE sample was analyzed at a concentration of 200 µg/mL. Based on the DMPBD calibration curve, the DMPBD concentration in the analyzed ESPE solution was calculated to be 19.82 µg/mL, corresponding to a DMPBD content of approximately 9.91% (*w*/*w*) in ESPE.

**Figure 6:**
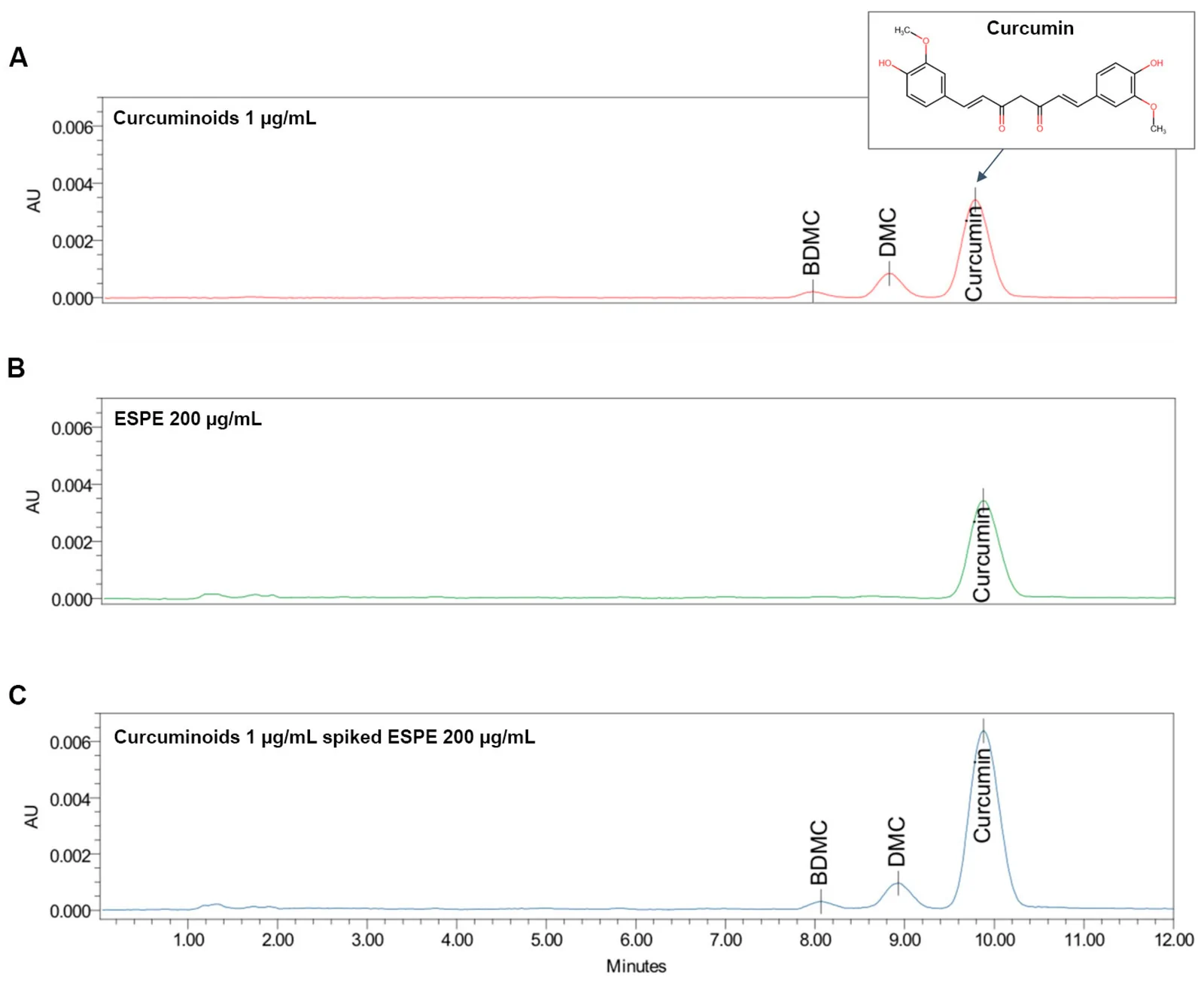
HPLC chromatograms of curcuminoid standards, ESPE, and the spiked sample. (**A**) Curcuminoid standard solution (1 μg/mL), showing the reference peaks used for compound identification; (**B**) ESPE (200 μg/mL) showing the endogenous curcumin peak; and (**C**) ESPE (200 μg/mL) spiked with curcuminoids (1 μg/mL), used for peak identity validation and recovery verification. Peaks: Bisdemethoxycurcumin (BDMC, *t_R_* = 8.065 min), Demethoxycurcumin (DMC, *t_R_* = 8.930 min), and Curcumin (*t_R_* = 9.882 min). *t_R_* = retention time.

HPLC analysis of curcuminoid standards, ESPE, and the spiked sample was performed to identify and quantify curcumin (Figure 6). Chromatographic separation was performed using a Waters 2695 HPLC system (Waters Corporation, Milford, USA) equipped with a Supelco Ascentis^®^ C18 column (Supelco, Bellefonte, USA) (150 mm × 4.6 mm, 5 µm). Isocratic elution was conducted at a flow rate of 1.0 mL/min using 2% aqueous acetic acid, acetonitrile, and methanol (50:45:5, *v*/*v*/*v*) with UV detection at 425 nm. The curcuminoid standard solution (1 µg/mL) showed three reference peaks corresponding to bisdemethoxycurcumin (BDMC; *t*_R_ = 8.065 min), demethoxycurcumin (DMC; *t*_R_ = 8.930 min), and curcumin (*t*_R_ = 9.882 min). The presence of curcumin in ESPE was confirmed by comparison of its retention time with that of the curcumin reference standard. The curcumin calibration curve showed excellent linearity (*R*^2^ = 0.9979). Quantification yielded a curcumin concentration of 0.976 µg/mL in the analyzed 200 µg/mL ESPE solution, corresponding to a curcumin content of approximately 0.49% (*w*/*w*) in ESPE.

### 2.4. The Microbial Profiles of Adult Zebrafish in Control and ESPE-Treated Groups

**Figure 7:**
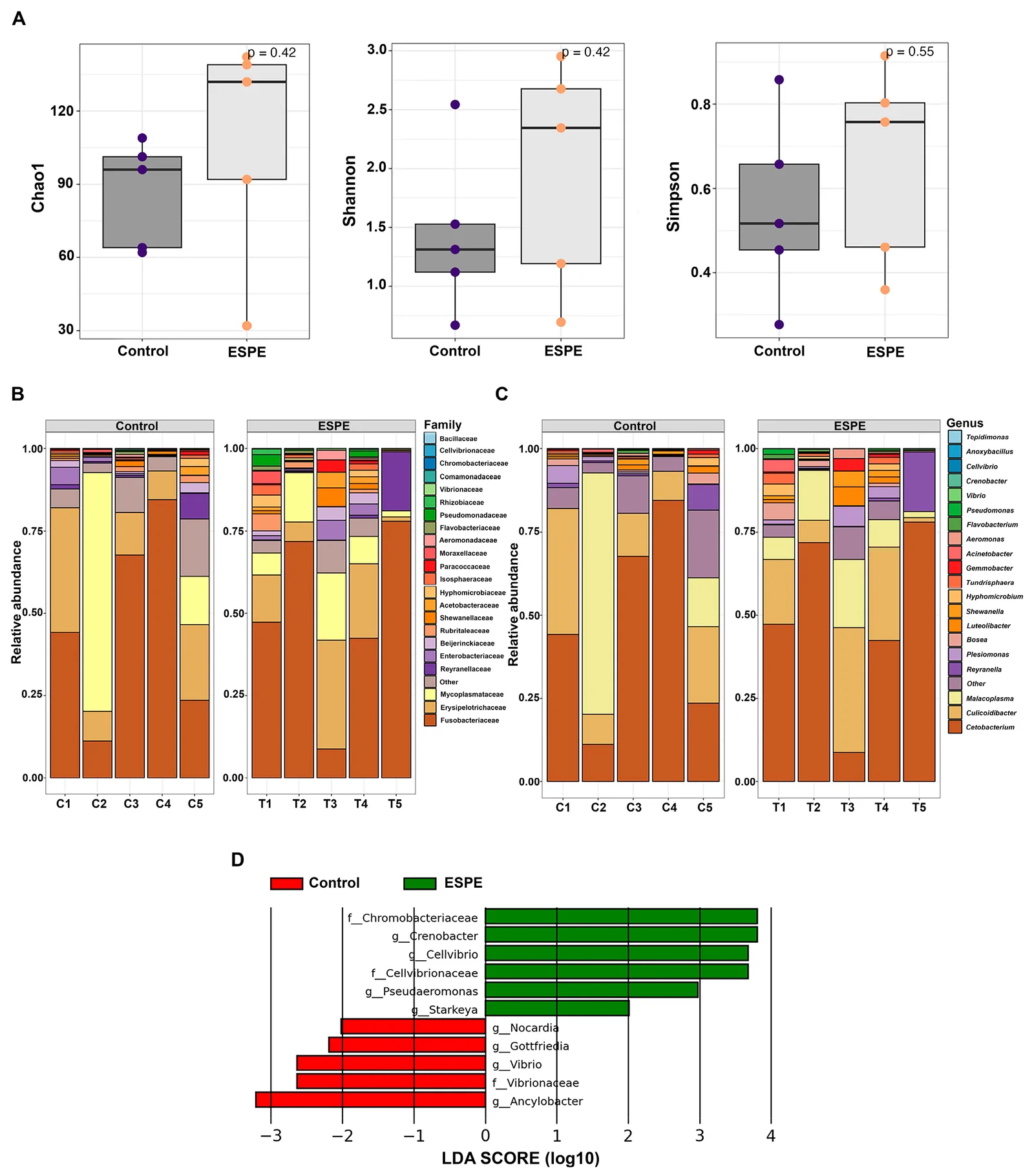
Microbial communities in the intestines of adult zebrafish were compared between ESPE-supplemented (*n* = 5) and control groups (*n* = 5). (**A**) The alpha diversity indices (Chao1, Shannon and Simpson). The relative abundances of the top 10 predominant microbes are presented at both the (**B**) family and (**C**) genus levels, each bar depicts the microbial composition for an individual sample. (**D**) Highlights intestinal microbes that were significantly more abundant in either the control or ESPE groups, as determined by LEfSe analysis (*p* < 0.05). ESPE: adult zebrafish fed an ESPE-enriched diet containing 20 mg ESPE/g (2% *w*/*w*).

Figure 7 displays the microbial composition and diversity within the zebrafish gut microbiome. Amplicon sequencing identified 785 unique Amplicon Sequence Variants (ASVs) across all samples, representing 232 genera. Analysis of alpha diversity, using Chao1 (*p* = 0.42), Shannon (*p* = 0.42), and Simpson (*p* = 0.55) indices, showed marginally higher values in the ESPE-treated group, though these differences were not statistically significant (Figure 7A). Similarly, no significant differences were observed in either alpha or beta diversity between the ESPE and control groups (PERMANOVA testing for beta diversity, *p* = 0.468; Figure S1 and Table S1). Taxonomic analysis at the phylum level revealed Fusobacterium as the predominant phylum in both groups (46.23% in controls, 49.66% in ESPE-treated; Figure S2). At the family level, Fusobacteriaceae was the most abundant, followed by Erysipelotrichaceae, with respective proportions of 49.66% and 10.43% in ESPE-treated fish and 46.23% and 18.31% in controls (Figure 7B). At the genus level, the dominant taxa were largely consistent between groups, although *Cetobacterium* was slightly more prevalent in the ESPE group (49.66% vs. 46.23%), while *Culicoidibacter* was less abundant (10.43% vs. 18.31%) (Figure 7C).

### 2.5. Distinct Alterations in Gut Microbiota Composition Between Control and ESPE-Supplemented Groups

Differential abundance analysis identified key genera in the intestinal microbiome of zebrafish under ESPE treatment. At the genus level, five genera showed significant differences (Figure 7D). Four genera, *Crenobacter* (*p* < 0.05, LDA = 4.13), *Cellvibrio* (*p* < 0.05, LDA = 3.96), *Pseudaeromonas* (*p* < 0.05, LDA = 3.25), and *Starkeya* (*p* < 0.05, LDA = 4.39), were significantly more abundant in the ESPE-treated group. Conversely, *Ancylobacter* (*p* < 0.05, LDA = 3.50), *Vibrio* (*p* < 0.05, LDA = 2.90), *Gottfriedia* (*p* < 0.05, LDA = 2.37), and *Nocardia* (*p* < 0.05, LDA = 2.05) were significantly enriched in the control group.

## 3. Discussion

*Z. purpureum*, or Phlai, has been extensively studied for its traditional medicinal uses and bioactive constituents [6,8,43,44]. However, its specific impacts on intestinal morphology, mucosal barrier integrity, and gut microbial composition have remained unexplored. Present evidence suggests that this study provides the first empirical evaluation of dietary *Z. purpureum* extract modulating host gut microbiota composition. Specifically, we evaluated the effects of ESPE across zebrafish life stages, focusing on developmental toxicity in embryos and larvae, alongside intestinal histology and microbiome profiling in adult zebrafish.

The noticeable dose-dependent phenotypic shift in developmental toxicity and morphological abnormalities in zebrafish embryos exposed to various concentrations of ESPE, as revealed by the spatiotemporal matrix, indicates a classical toxicological threshold effect [45]. At lower concentrations (10–25 µg/mL), the embryonic defense mechanisms are sufficient to prevent acute mortality, shifting the pathology toward sublethal structural variations [45,46]. However, exceeding the critical threshold (>50 µg/mL) overrides the embryo’s physiological buffering capacity, triggering widespread, rapid systemic failure (coagulation and late coagulation) [45]. This aligns with standard toxicological models, in which natural extracts contain bioactive molecules that act as therapeutic agents at low doses but shift to cytoprotective-disrupting agents at elevated doses [46].

Our study’s core lethal endpoints align with OECD Test Guideline 236 for Fish Embryo Acute Toxicity (FET): (1) Protein coagulation within 24–72 h post-fertilization (hpf) indicates severe necrosis or oxidative stress from high ESPE doses; (2) Lack of tail detachment across 24–72 hpf reveals ESPE disrupts signaling pathways and apoptosis needed for body axis extension and tissue remodeling [47]; (3) Sublethal cardiovascular toxicity and edema are shown by blood congestion and pericardial edema at 72–96 hpf, suggesting endothelial barrier disruption or osmotic imbalance [47,48]. Blood congestion may signal decreased cardiac output or microvascular blockage [47]; (4) Regenerative potential: localized, gradually developing effects at sub-lethal concentrations suggest a window for tissue repair (endothelial proliferation or fluid regulation) if the toxicant is metabolized or removed [45,46,47]. By 96 hpf, ESPE exposure leads to irreversible skeletal and neurological defects in zebrafish embryos, notably spinal curvature (kyphosis) from neuromuscular toxicity or notochord cell death [49]. Deformed otoliths, critical for balance and hearing, indicate ESPE disrupts calcium carbonate formation or inner-ear signaling [47]. Craniofacial malformations suggest impaired neural crest cell migration and differentiation, which are essential for the development of the jaw and facial cartilage [47,48]. These effects follow a classic biphasic, dose-dependent pattern: low doses are tolerated, but high doses cause permanent damage; such patterns are common in botanical extracts: low doses protect and high doses overwhelm redox balance [45,46].

Histological analysis revealed that ESPE-treated adult zebrafish maintained normal, well-organized muscle architecture with intact cellular boundaries and no collagenous fibrosis. ESPE exposure increased the density of smaller muscle fibers, indicating a shift toward a juvenile or regenerative profile while preserving muscle quality similar to controls. Smaller myofibrils with clear boundaries are markers of active muscle regeneration, in contrast to the disorganized structure observed in zebrafish muscular dystrophy models [50]. This suggests ESPE supports structural stability and cellular remodeling. These results highlight zebrafish as a model for muscle aging and disease, showing ESPE’s potential to counter muscle degradation [51]. ESPE also caused epidermal thinning and a reduction in the size and number of goblet cells in adult zebrafish skin. While such changes might imply a weakened barrier, they resemble the naturally thin, less-stratified skin of juvenile zebrafish [52]. The reduction in goblet cells similarly reflects a juvenile-like state rather than damage. These cutaneous changes, together with the muscle findings, indicate ESPE promotes an adaptive, regenerative phenotype without harming core tissue structures. The skin’s anatomical framework, including dermal scales and melanophore distribution, remained intact. This dual induction of juvenile-like features in muscle and skin suggests ESPE modulates tissue homeostasis toward regeneration, underscoring its potential for biomedical and dermatological applications [52,53]. This regenerative and adaptive phenotype encompasses both external and internal features, notably enhancing mucosal immunity and gut health. In ESPE-treated zebrafish, intestinal histology shows significantly improved gut condition, as indicated by an increased number of goblet cells and better villus architecture [54,55]. Although Paneth cells were not clearly identified, a slight rise in immune-related cells suggests mild yet beneficial immune modulation [54]. Overall, these findings highlight the treatment’s capacity to enhance mucosal function and maintain gut homeostasis in zebrafish.

The intestinal microbiome analysis in zebrafish highlighted statistically significant differences in microbial genera between groups, with specific genera enriched in the ESPE-treated group. This suggests that the ESPE-treated selectively promoted beneficial microbes while limiting less favorable ones. Several herbal studies reported modulation of the gut microbiome and associations with herbal treatment [56,57,58]. The use of herbal remedies for their health benefits, along with the interplay between natural compounds and the gut microbiome, underscores the crucial role of gut microbial composition and function in supporting overall animal health [56]. The ESPE-treated group showed a higher proportion of beneficial or commensal genera, potentially enhancing gut health and resilience. Differential analysis revealed that the gut microbiome comprised diverse taxa with distinct functional roles. The ESPE-treated group was enriched with bacteria known for their beneficial properties. Family Chromobacteriaceae produce antimicrobial compounds that help maintain gut balance by suppressing pathogens [59]. Genus *Crenobacter* is recognized as a phosphate-solubilizing bacterium and is reported to metabolize a wide range of organic substrates involved in carbohydrate and lipid metabolism [60,61]. This metabolic versatility suggests adaptation to diverse environmental conditions. Furthermore, the species *Crenobacter intestini* sp. was recently isolated from *Konosirus punctatus*, suggesting a potential role in nutrient cycling within aquatic microbial communities [61,62]. *Cellvibrio* species enzymatically degrade complex polysaccharides, promoting fiber breakdown and generating short-chain fatty acids that support the host’s intestinal stability and health [63]. The genus *Pseudomonas* becomes enriched during the feed domestication of *Aplodinotus grunniens*, contributing to feed efficiency and supporting the host cellular homeostasis [64]. Genus *Starkeya*, a nitrogen-fixing bacterium, participates in nitrogen cycling and offers metabolic benefits to the gut, although its health effects remain unclear [65]. Additionally, the control group was dominated by other genera, such as *Nocardia*, commonly found in soil, which can degrade organic compounds; while some strains are opportunistic pathogens, many have a limited impact on gut health [66]. The genus *Gottfriedia* is thought to be part of the normal flora, and some species, including *Gottfriedia endophyticus* sp., have been reported to metabolize indole to produce acetic acid [67]. The genus *Vibrio* includes both pathogenic and commensal species; non-pathogenic *Vibrio* are common in aquatic and possibly gut environments, underscoring the need to distinguish between strains in microbiome studies [68]. Genus *Ancylobacter*, commonly associated with soil and water, may enter the gut via environmental exposure, but its function within the gut is largely unknown [69].

Rather than broadly disrupting the core gut microbiota, ESPE modulates specific taxa while preserving community stability. ESPE selectively enriches microbiota, *Cellvibrio* (polysaccharide degradation to short-chain fatty acids) and *Crenobacter* (carbohydrate/lipid processing and phosphate solubilization), hypothesized to enhance mucosal energy harvest and nutrient solubilization [60,61,62,63]. Concurrently, the selective enrichment of antimicrobial-producing Chromobacteriaceae and homeostatic *Pseudomonas* strengthens barrier defense, restricting potential opportunistic taxa such as *Nocardia* and *Vibrio* species [59,66,68]. Minor shifts in specialized environmental guilds (*Starkeya* and *Ancylobacter*) reflect localized metabolic adaptations in nitrogen and nutrient cycling rather than community destabilization, suggesting that ESPE supports metabolism and colonization resistance while maintaining core microbiome homeostasis [65,69]. However, sample size constraints limit the statistical power of our microbiome analysis. Increasing the cohort size in future studies will provide the robustness required to detect significant differences in core gut microbiota diversity. In addition, future in-depth functional investigations, such as metagenomics or metabolomics, will provide a deeper understanding of the precise biological mechanisms underlying ESPE use.

In the present study, quantitative chromatographic profiling demonstrated that DMPBD, a prominent phenylbutanoid derivative, constitutes approximately 9.91% (*w*/*w*) of the ESPE, corresponding to a concentration of 19.82 µg/mL in the analyzed solution. In contrast, curcumin was detected at a minor concentration of approximately 0.49% (*w*/*w*) or 0.976 µg/mL, indicating that curcumin serves solely as a trace analytical marker rather than the primary driver of biological activity. Given that DMPBD is present at a concentration more than 20-fold higher than that of curcumin, the observed biological responses including developmental toxicity profiles, mucosal preservation, and gut microbiota modulation are predominantly attributable to the high abundance of phenylbutanoids such as DMPBD in ESPE, rather than curcuminoids.

Consequently, the precise molecular thresholds and potential synergistic interactions among these phenylbutanoids require further characterization. Notably, the sub-lethal ESPE concentration range of 10–25 µg/mL represents a promising therapeutic window that falls well below the LC50, demonstrating significant efficacy in preserving intestinal mucosal integrity and modulating gut microbiota without inducing developmental toxicity. To fully elucidate the underlying mechanisms, subsequent studies incorporating comprehensive biochemical assays, functional metagenomic profiling, and targeted pathway analyses are warranted. Furthermore, while the zebrafish serves as an excellent in vivo model for high-throughput screening, future mammalian studies will be essential to validate these gut health-modulating effects for potential biomedical applications.

The limitation of the present study is the exclusive utilization of female adult zebrafish to minimize sex-dependent variability during baseline toxicological and histological evaluations. While this design ensured high reproducibility, future research incorporating both male and female zebrafish will be necessary to investigate potential sex-dimorphic responses and fully elucidate the systemic safety profile of ESPE.

Finally, the present exposure study was executed as a single independent experimental trial using well-defined biological replicates per group. While internal sample sizes provided adequate statistical power for comparative evaluations, future independent replications will be valuable to further confirm long-term reproducibility across different experimental cohorts.

## 4. Materials and Methods

### 4.1. Preparation of the Ethanolic-Soluble Phlai Extract (ESPE)

The fresh Phlai rhizomes used in this study were obtained from plants sourced from LittlePark Farm, Bang Rak Noi, Mueang, Nonthaburi, Thailand. A voucher specimen was authenticated by Dr. Sunisa Sangvirotjanapat and has been deposited at the Herbarium of Mahidol University (PBM), Nakhon Pathom, Thailand, under voucher numbers PBM 006862-006863. To prepare the specimen, fresh rhizomes were processed in a 70 °C hot-air dryer according to the reference protocol. Then they were ground into fine powder; 10% (*w*/*v*) Phlai powder was mixed with 70% (*v*/*v*) ethanol [70]. This mixture was macerated at 30 °C for 3 h with constant shaking at 120 revolutions per minute (rpm). During extraction, the flask was covered with aluminum foil to block light. After maceration, the mixture was sequentially filtered through Whatman No. 3 and No. 1 filter papers to remove any deposits. The filtered solution was stored at −80 °C and, if necessary, thawed at −20 °C before further processing. The stored ethanolic Phlai extract was then concentrated by rotary evaporation in a 50 °C water bath, with the condenser set to 10 °C to ensure condensation. After evaporation, the concentrate was refrigerated and subsequently freeze-dried. For the feeding experiment, we prepared pellets for adult zebrafish by homogenizing 20 g of Hi-Grade 9006T diet (CPF, Bangkok, Thailand) with 0.4 g of ESPE to make an ESPE-supplemented diet at 20 mg/g (2% *w*/*w*). The mixture was then extruded into pellets for feeding, and the resulting supplemented diet was used as the experimental feed.

### 4.2. Animal Ethics and Institutional Approval

All experimental protocols involving aquatic animals were rigorously conducted in compliance with the ethical frameworks and policies mandated by Mahidol University and the Office of the National Research Council of Thailand (NRCT). The overarching study framework, animal husbandry protocols, and specific interventions were formally evaluated and approved by the Faculty of Science, Mahidol University-Institutional Animal Care and Use Committee (MUSC–IACUC), the Central Animal Facility (CAF), Faculty of Science, Mahidol University, under the designated Protocol Number: MUSC68-003-773.

### 4.3. Zebrafish Maintenance and Husbandry

Clinically healthy adult female zebrafish (*Danio rerio*), aged between 6 months and 1 year, with a body weight ranging from 0.5 to 1 g, were procured from the licensed ornamental fish and aquarium plant wholesale market under the ornamental fish and aquatic plants embryo transfer and culture technology research and development center of the Department of Fisheries, Pathumthani, Thailand. Adult female zebrafish were selectively utilized for the adult exposure assays to reduce physiological, metabolic, and hormonal variations associated with sex-specific differences, thereby ensuring baseline consistency and optimal reproducibility across experimental groups. Upon arrival at the facility, the fish underwent a mandatory 14-day isolation period in a dedicated quarantine aquarium. This measure enabled intensive daily screening to rule out potential clinical abnormalities or infectious manifestations, thereby establishing an optimal baseline health status before experimental assignment.

The aquatic cohorts were subsequently housed in independent glass aquaria, each with a 5.4 L capacity and physical dimensions of 12 cm × 25 cm × 18 cm. These housing units were seamlessly linked to a closed-loop recirculating water architecture equipped with robust continuous filtration and constant mechanical aeration to remove metabolic debris and maximize water quality. A systematic stocking density was enforced at 16 fish per aquarium, equivalent to roughly 3–6 fish per liter of water volume. Physical and chemical parameters of the microenvironment were monitored daily, maintaining a stable thermal environment at 25–27 °C and a consistent pH range of 7.4–7.8. Critical water chemistry metrics, including dissolved oxygen (DO), total ammonia, and nitrite concentrations, were systematically verified weekly. Standard dechlorinated tap water, conditioned in an open auxiliary reservoir for several days to facilitate the natural clearance of gaseous chlorine, was used for a scheduled 50% weekly water exchange. The environmental lighting regimen relied on conventional fluorescent lighting operating under a standardized 14 h light and 10 h dark cycle. Throughout both the pre-conditioning and formal testing windows, the subjects received a commercial flake diet (Hi-Grade 9006T, CPF, Thailand) administered twice daily.

### 4.4. Zebrafish Propagation and Embryo Harvesting

To generate the necessary biological material for subsequent embryotoxicity profiling, parental zebrafish were selected based on peak vigor and reproductive maturity. Breeding pairs comprising roughly a female:male ratio of 1:2 were introduced into a shared spawning tank just before nightfall. The floor of this specialized breeding chamber was fitted with a protective aluminum mesh grid. This barrier design was vital for shielding newly deposited eggs and successfully preventing adult fish from engaging in post-spawning cannibalism. Spontaneous mating and natural fertilization events were triggered by the onset of light exposure the following morning. Immediately after the presence of freshly fertilized eggs was confirmed, the parental fish were carefully extracted and returned to their respective home aquaria, thereby ensuring the collection of tightly synchronized developmental cohorts. Healthy, naturally spawned embryos were delicately collected from the tank floor, thoroughly washed, and meticulously evaluated under a stereomicroscope to harvest viable specimens at 3–4 h post-fertilization (hpf) for the acute toxicity treatments.

### 4.5. Fish Embryo Acute Toxicity (FET) Test

The acute toxic effects and sublethal developmental impacts of ESPE were evaluated using the FET test [71] in accordance with OECD Test Guideline 236 [72]. Viable zebrafish embryos at 3-4 hpf were placed individually in 96-well plates containing 200 μL of treatment solution per well. Embryos were exposed to ESPE at 10, 25, 50, 75, and 100 μg/mL. Final treatment solutions were prepared by diluting ESPE stock with 10% Hanks’ balanced salt solution (HBSS). The final ethanol concentration was 0.35% (*v*/*v*) in all treatment wells, and the control group received 0.35% (*v*/*v*) ethanol in *HBSS* without ESPE to control for potential vehicle effects. This concentration remains far below the developmental toxicity threshold (1.25–1.5% *v*/*v*) reported applied for both zebrafish embryonic and adult models in Section 4.6 [73]. Exposures lasted up to 96 hpf, with treatments replaced every 24 h. Morphological assessments were performed at 24, 48, 72, and 96 hpf using a stereomicroscope to monitor toxicity. Acute lethal toxicity was determined based on the four OECD TG 236 endpoints: egg coagulation, lack of somite formation, lack of tail-bud detachment, and no heartbeat. Sublethal abnormalities, such as pericardial edema, blood congestion, kyphosis, and otolith and craniofacial malformations, were screened to establish extract safety. Changes were observed and photographed using an inverted microscope at 10× magnification.

### 4.6. Adult Zebrafish with ESPE Supplementation

The 6-month-old adult female zebrafish were separated into control (*n* = 8) and treatment groups (*n* = 8). The control group received a standard diet (Hi-Grade 9006T, CPF, Thailand), whereas the treatment group received 20 mg ESPE/g diet (2% *w*/*w*) once daily. This concentration was selected based on previous studies showing it does not cause acute toxicity in adult zebrafish. Feeding continued until the zebrafish reached satiation within 5 min, at which point the feeding session ended [74]. The animal’s health was monitored and recorded daily for 14 days. For microbiome analysis, five zebrafish from each condition were selected and euthanized using 200 µg/mL of ethyl 3-aminobenzoate for the euthanasia. Three zebrafish were dissected, and organs such as the intestine and skeletal muscle were collected for histopathological investigation.

### 4.7. Tissue Processing and Staining

Adult zebrafish were fixed in 10% buffered formalin and then decalcified in 0.5 M ethylenediaminetetraacetic acid (EDTA) at pH 7.8 for 7 days. The organs were collected for tissue processing with 70%, 95%, and 100% of EtOH for 2 h. per each concentration, followed by alcohol with xylene to make the tissue more translucent. The embedding surrounded the samples by a medium wax (Thermo Fisher Scientific, Kalamazoo, MI, USA), which provides sufficient external support during sectioning. For the sectioning part, cut the sample into thin sections of 5 µm. The tissue samples were stained with hematoxylin and eosin (H&E) for microscope investigation. To evaluate extracellular matrix remodeling, connective tissue architecture, and tissue fibrosis, Masson’s Trichrome Staining (MTS) was performed using a Trichrome Staining Kit obtained from the Histopathology Laboratory (Department of Pathobiology, Faculty of Science, Mahidol University) with protocols. The procedure was carried out briefly as follows: following deparaffinization and rehydration, the tissue sections were immersed in Bouin’s fixative overnight at room temperature to facilitate chromatin mordanting and to optimize subsequent staining differentiation. The sections were then incubated in Weigert’s iron hematoxylin for 10 min to selectively label cell nuclei a dark blue-black, followed by a thorough rinse in running tap water. Next, the sections were immersed in Biebrich scarlet-acid fuchsin solution for 4 min to impart a vibrant red coloration to the cytoplasm and muscle fibers. The dermal collagen fibers were subsequently counterstained by immersion in a light green solution for 7 min, rendering collagen-rich structures distinctly blue-green and highly distinguishable from the red-stained cytoplasmic and myofiber components [75]. Post-staining differentiation was performed in 1% acetic acid to maximize the contrast and structural definition between these distinct tissue elements. Finally, the stained sections were dehydrated through a graded ethanol series, cleared in xylene, and cover slipped using a resinous mounting medium (Fisher Chemical^TM^, Fair Lawn, NJ, USA). This optimization allowed for high-contrast digital segmentation of the blue-green collagen network against the red cellular matrix of muscle fibers. Digital microphotographs were captured using a light microscope (CX33, Olympus, Tokyo, Japan, and DG-105-W; CANOSCOPE^®^ 5 MP, FHD Digital Camera, Shenzhen, China) of the target organs, specifically the skin (upper dorsal head region, starting immediately where the rigid bony facial shield ends) and the mid-intestinal mucosa. To ensure representative sampling and avoid repeated cellular counting during quantitative analysis, 3 non-consecutive longitudinal sections per specimen (*n* = 3 per group) were collected at an inter-section distance of 50 µm. For each organ per individual fish, 3 randomized, non-overlapping high-power field images were captured and analyzed to evaluate morphological and quantitative histological differences between the control and ESPE-treated groups.

### 4.8. Quantitative Phytochemical Analysis by HPLC

#### 4.8.1. Determination of (E)-1-(3,4-dimethoxyphenyl)butadiene (DMPBD)

The major phenylbutanoid constituent was quantified using high-performance liquid chromatography (HPLC). The ESPE was dissolved in methanol, sonicated in an ultrasonic bath for 5 min to ensure complete dissolution, and filtered through a VertiClean™ Nylon syringe filter (Vertical Chromatography Co., Ltd., Bangkok, Thailand) (13 mm, 0.45 µm pore size) prior to injection. Chromatographic separation was achieved on a SunFire™ C18 column (Waters Corporation, Wexford, Ireland) (150 mm × 4.6 mm, 5 µm) at room temperature. Isocratic elution was conducted at a flow rate of 1.0 mL/min using a mobile phase consisting of acetonitrile and 2% aqueous acetic acid (52:48, *v*/*v*). The injection volume was set to 20 µL, and UV detection was monitored at a wavelength of 284 nm. A calibration curve was constructed using (E)-1-(3,4-dimethoxyphenyl)butadiene; DMPBD standard (CAS No. 75560-74-6; Toronto Research Chemicals, Vaughan, ON, Canada) across a concentration range of 0–80 µg/mL (1, 2, 5, 10, 20, 40, and 80 µg/mL).

#### 4.8.2. Analysis of Curcuminoid Reference Markers

To evaluate trace curcuminoid content, the prepared ESPE sample solution was analyzed using a Waters 2695 HPLC system (Waters Corporation, Milford, MA, USA) equipped with a Supelco Ascentis^®^ (Supelco, Bellefonte, PA, USA) C18 column (150 mm × 4.6 mm, 5 µm) maintained at room temperature. Isocratic elution was performed at a flow rate of 1.0 mL/min with an injection volume of 10 µL. The mobile phase comprised 2% aqueous acetic acid, acetonitrile, and methanol (50:45:5, *v*/*v*/*v*/*v*). Detection was set at a wavelength of 425 nm. A calibration curve for curcumin was established using standard curcumin powder from *Curcuma longa* (1,7-Bis(4-hydroxy-3-methoxyphenyl)-1,6-heptadiene-3,5-dione; CAS No. 458-37-7; Sigma-Aldrich, St. Louis, MO, USA) over a concentration range of 0.5–25 µg/mL. Under these conditions, retention times for bisdemethoxycurcumin (BDMC), demethoxycurcumin (DMC), and curcumin were observed at 8.07, 8.93, and 9.88 min, respectively.

### 4.9. Intestinal Microbiome Analysis

Genomic DNA from zebrafish intestines was extracted with the QIAamp Fast DNA Stool Mini Kit (QIAGEN, Hilden, Germany) according to the manufacturer’s instructions. Extracted DNA samples were submitted to NovogeneAIT Genomics (Singapore) for sequencing. The V3V4 region of the 16S rRNA gene was amplified using universal primers 341F (5′-CCTAYGGGRBGCASCAG-3′) and 806R (5′-GGACTACNNGGGTATCTAAT-3′), followed by paired-end sequencing on the Illumina NovaSeq 6000 system (InstrumentID A01426) (San Diego, CA, USA). Sequence data are available in NCBI under BioProject PRJNA1477803. The quality of 16S rRNA amplicon sequences from zebrafish intestines was initially evaluated using FastQC (version 0.11.9) [76]. Cutadapt (version 4.0) was used to remove PCR primers, Illumina index adapter sequences, and low-quality regions from the sequencing data [77]. Subsequent analyses were performed with the QIIME2 (version 2024.5) pipeline following quality control and removal of primers and adapters [49]. During DADA2 preprocessing, low-quality reads were filtered out, sequencing errors were corrected through denoising, and genuine biological sequences were identified. The denoised forward reads were then merged with the reverse complement of the corresponding reverse reads to form contig sequences. Chimeric sequences were identified and removed. The representative sequences generated in QIIME2 were then taxonomically classified using the SILVA 138.1 reference database [78]. To visualize the relative abundances of the top 10 taxa at the phylum and genus levels, stacked bar charts were generated. Significant differences in taxa abundance between the control and ESPE groups were identified using Linear Discriminant Analysis Effect Size (LEfSe) analysis (version 1.1.2) [79]. Taxa were considered significantly different if they had an LDA score of at least 2 and an unadjusted *p*-value ≤ 0.05.

### 4.10. Statistical Analysis

An independent-samples *t*-test was performed to assess differences in goblet cell counts in both the skin and intestine between control and ESPE groups, applying a significance level of *p* < 0.05. Alpha diversity, representing within-sample microbial variation, was measured using the Chao1, Shannon, and Simpson indices. Statistical comparisons of diversity indices between groups were carried out using the *t*-test function in the vegan package (*p* < 0.05) [80]. Beta diversity, reflecting differences in microbial communities between samples, was evaluated using Principal Coordinates Analysis (PCoA) based on Bray–Curtis dissimilarities. Pairwise differences in community composition were assessed with Permutational Multivariate Analysis of Variance (PERMANOVA) [81] utilizing the adonis 2 function from the vegan package. All statistical analyses were conducted in RStudio (version 4.0.2) [82].

## 5. Conclusions

This study provides evidence that ethanolic-soluble Phlai extract (ESPE), standardized using DMPBD and curcumin as chemical markers, produces measurable biological effects in zebrafish. The observed intestinal histological features and absence of major systemic tissue abnormalities under the tested conditions suggest that ESPE warrants further investigation for its potential effects on intestinal health. Although overall gut microbial alpha and beta diversity indices remained unchanged, exploratory differential abundance analysis indicated differences in selected microbial genera following ESPE treatment. These taxon-level findings should be considered hypothesis generating rather than evidence of a beneficial microbiome modulation. The dose-dependent responses observed in this study further emphasize the importance of dose optimization and safety assessment. Collectively, the findings support continued preclinical investigation of ESPE, particularly to clarify its intestinal effects, microbiological relevance, dose-response relationships, and short-term safety before considering potential translational applications.

## Data Availability

All data produced or examined throughout this study have been incorporated into this published article and Supplementary Materials. Sequence data are available in NCBI under BioProject PRJNA1477803. Further inquiries can be directed to the corresponding author.

## Supplementary Materials

The following supporting information can be downloaded at: https://www.mdpi.com/article/10.3390/ijms27198684/s1.
