# Supplementary material for: Toxicological Evaluation of Zingiber purpureum Extract and Its Effects on Gut Microbiota in Zebrafish
Source: Int J Mol Sci. 2026 Sep 28;27(19):8684. doi: 10.3390/ijms27198684 (PMC13664765; doi:10.3390/ijms27198684)
Supplement: Supplementary file 1 [file ijms-27-08684-s001.zip › ijms-4478462-supplementary.pdf]

Supplementary

Supplementary Table S1. PERMANOVA testing for the dissimilarity of zebrafish intestinal microbiome between the control and ESPE-treated groups based on Bray-Curtis distance.

	Df	SumOfSqs	R^2	F	Pr(>F)
Condition	1	0.1274	0.09187	0.8093	0.468
Residual	8	1.2594	0.90813		
Total	9	1.3868	1.00000		

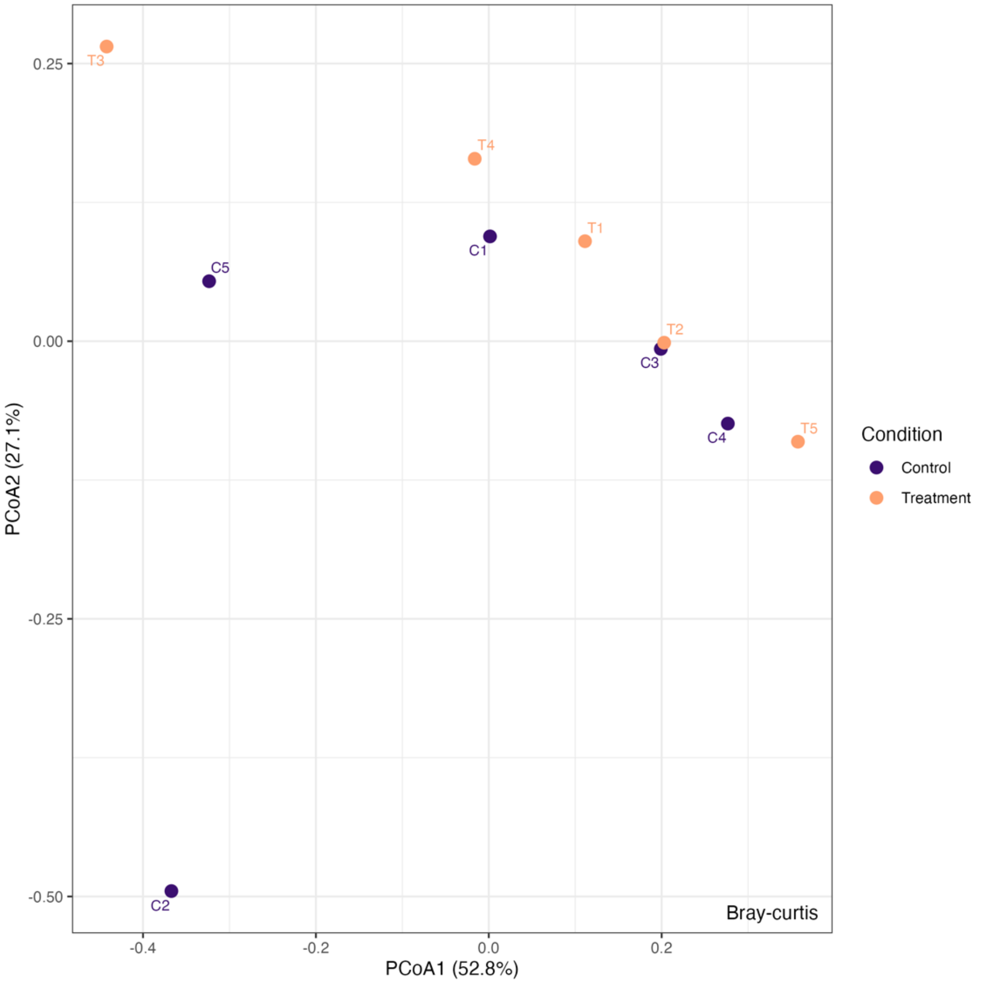

Supplementary Figure S1. The Principal Coordinates Analysis (PCoA) plot on the zebrafish intestinal microbiome of control and ESPE-treated groups based on Bray-Curtis distance.

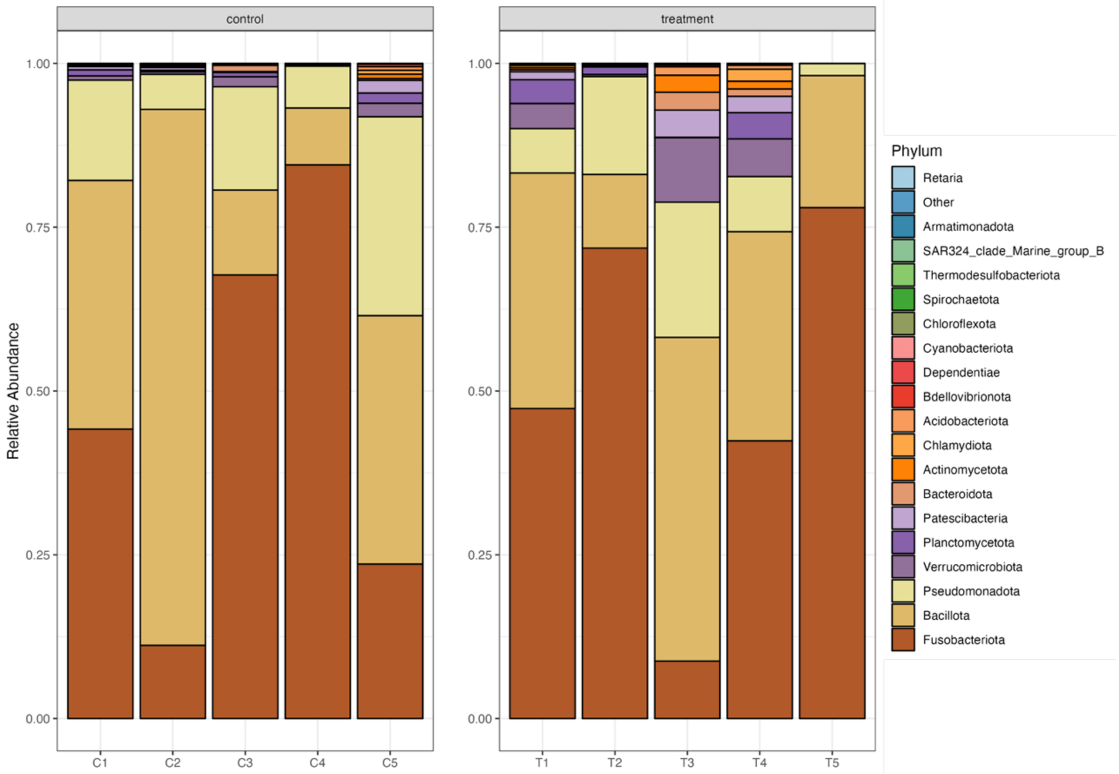

Supplementary Figure S2. Microbial profiles in the intestine of adult zebrafish in response to ESPE supplementation and the control group, the compositions of the top 10 most abundant microbes are shown at the phylum level.
